# Multi‐Omics Integration Identifies a Five‐Gene Metabolic Signature With Experimental Validation in Clear Cell Renal Cell Carcinoma

**DOI:** 10.1002/cnr2.70673

**Published:** 2026-09-06

**Authors:** Yi Peng, Suobing Qin, Xiwen Li, Lijun Wan, Rijian Guan

**Affiliations:** ^1^ Department of Urology The People's Hospital of Danyang; Affiliated Danyang Hospital of Nantong University Danyang China; ^2^ Department of Urology The Quzhou Affiliated Hospital of Wenzhou Medical University, Quzhou People's Hospital Quzhou China

**Keywords:** clear cell renal cell carcinoma, metabolic reprogramming, precision medicine, prognostic model, tumor microenvironment

## Abstract

**Background:**

Clear cell renal cell carcinoma (ccRCC) is hallmarked by profound metabolic reprogramming; however, its intricate crosstalk with the tumor immune microenvironment (TIME) and its clinical ramifications remain inadequately elucidated. This study aims to systematically decipher the metabolic‐immune interplay in ccRCC through multi‐omics integration, with the goal of identifying robust prognostic biomarkers and actionable therapeutic vulnerabilities.

**Aims:**

This study aims to systematically decipher the metabolic–immune interplay in clear cell renal cell carcinoma (ccRCC) through multi‑omics integration, and to identify robust prognostic biomarkers and actionable therapeutic vulnerabilities that can inform precision risk stratification and individualized treatment strategies.

**Methods:**

We integrated bulk transcriptomic, genomic, and clinical data from multiple ccRCC cohorts. Differential expression and functional enrichment analyses were performed to characterize metabolic pathway alterations. Mendelian randomization (MR) was employed to infer causal relationships between metabolic disorders and ccRCC risk. A machine learning‐based prognostic framework, incorporating SHAP (SHapley Additive exPlanations) for feature interpretability, was constructed and rigorously validated. TIME heterogeneity was dissected using deconvolution algorithms, while drug sensitivity, tumor mutation burden (TMB), and TIDE scores were utilized to assess therapeutic responses and immune evasion. Candidate gene function was evaluated through in vitro gain‐ and loss‐of‐function assays, with expression validated via TCGA, HPA, western blot, and qRT‐PCR.

**Results:**

Enrichment analysis identified coordinated dysregulation in lipid metabolism, energy homeostasis, and hypoxia response pathways. MR analysis confirmed lipid metabolism disorders as a causal risk factor for ccRCC. Our machine‐learning model, centered on five core SHAP‐identified features (SUCLA2, ACAT1, PC, SUCLG1, and HMGCS2), demonstrated superior predictive accuracy over conventional clinical staging. Immune profiling unveiled dichotomous TIME states: the low‐risk group retained active immune surveillance, whereas the high‐risk group was enriched with immunosuppressive subsets. Drug sensitivity screening pinpointed LY2109761 and carmustine as high‐risk‐specific candidate agents. Furthermore, TMB and TIDE analyses stratified high‐risk patients displaying genomic instability and immune evasion phenotypes. Functionally, SUCLA2 knockdown significantly enhanced ccRCC cell proliferation and invasion, while its overexpression suppressed these malignant phenotypes, corroborating its tumor‐suppressive role. Expression patterns of the hub genes were consistently validated across multi‐level datasets and experimental assays.

**Conclusion:**

This study establishes a precision oncology framework for ccRCC by functionally linking metabolic biomarkers, immunophenotypes, and stratified therapeutic strategies. Importantly, we identify SUCLA2 as a potential functional tumor suppressor and a promising target for further mechanistic and translational investigation.

## 1 Introduction

Clear cell renal cell carcinoma (ccRCC) is the most common and invasive subtype of kidney cancer, accounting for approximately 75% of cases, with a low 5‐year survival rate for metastatic disease [1]. In recent years, advances in diagnostic technology have improved the early detection rate of RCC. Extracellular vesicles have become noninvasive biomarkers for RCC diagnosis and monitoring due to their stable mRNA and miRNA contents [2]. One hallmark of ccRCC is its profound metabolic reprogramming, driven by the loss of VHL genes and subsequent stabilization of HIF‐1α, which coordinates the transition toward glycolysis, lipid accumulation, and amino acid metabolism dysregulation [3, 4, 5]. Indeed, the loss of functional VHL protein is considered a defining event in clear cell RCC development, and the principal downstream oncogenic mechanism appears to be HIF‐2α accumulation and constitutive HIF transcription factor activity. Notably, belzutifan is a potent small‐molecule inhibitor of HIF‐2α that prevents heterodimerization with HIF‐1β into an active transcription factor and has shown activity in clear cell RCC. A recently published Phase III study demonstrated a significant benefit of belzutifan over everolimus concerning progression‐free survival and objective response in patients with advanced ccRCC who had previously received immune checkpoint and antiangiogenic therapies [6]. Although these metabolic changes are well documented, little is known about their dynamic interactions with the tumor immune microenvironment (TIME) and their collective impact on prognosis and treatment response. This study utilizes a multi‐omics framework to analyze the metabolic immune axis in ccRCC, integrating transcriptomics, genomics, and pharmacogenomics to identify potential biomarkers and treatment vulnerabilities.

Metabolic reprogramming in ccRCC surpasses the Warburg effect, including lipid droplet accumulation, mitochondrial dysfunction, and abnormal nitrogen metabolism [7, 8]. Recent studies have emphasized the role of branched‐chain amino acid (BCAA) catabolism in promoting tumor growth through mTORC1 activation [9], and dysregulated fatty acid oxidation promotes immune escape by inhibiting dendritic cell function [10]. Despite these advances, most research has focused on isolated metabolic pathways and overlooked the systematic integration of metabolic dysregulation and immune evasion mechanisms. Moreover, current prognostic models for ccRCC heavily rely on clinical staging and lack metabolic‐immune integration. This limitation could be addressed by incorporating novel molecular markers such as long noncoding RNAs (lncRNAs). The total number of lncRNAs is increasing due to more sensitive detection methods and is now greater than the sum of all protein‐coding genes. lncRNAs are mainly transcribed by RNA polymerase II, undergo posttranscriptional modifications, and have been detected in the nucleus, nucleolus, cytoplasm, and mitochondria. Accumulating evidence suggests a mechanistic link between cancer and lncRNA dysregulation, making lncRNA molecules potential therapeutic targets and biomarkers [11]. Here, we use differential expression and enrichment analysis to map the global metabolic pattern of ccRCC, revealing the coordinated dysregulation of lipid metabolism, energy homeostasis (TCA cycle), and hypoxia adaptation. Importantly, Mendelian randomization (MR) analysis established a causal relationship between lipid metabolism disorders and ccRCC risk, providing genetic evidence for metabolic intervention strategies.

Emerging evidence suggests that metabolic reprogramming directly shapes TIME. For example, lactate secretion from glycolytic tumors can inhibit cytotoxic T cell activity [12]. The accumulation of cholesterol in myeloid‐derived suppressor cells (MDSCs) enhances immune suppression [13]. In ccRCC, metabolic immune crosstalk has not been fully explored, especially in the context of risk stratification. Our immune deconvolution analysis revealed different TIME states: low‐risk tumors exhibited preserved immune surveillance (enriched resting CD4^+^ T cells and dendritic cells), while high‐risk tumors were characterized by increased immune suppression subgroups (Treg and T follicular helper cells) and checkpoint subsets. These findings are consistent with our SHapley Additive exPlanations (SHAP)‐based prognostic model, which identifies SUCLA2 and HMGCS2 as top predictive features, suggesting their important roles in metabolic disorders and immune regulation.

At present, the prognostic model of ccRCC heavily relies on clinical staging and lacks metabolic‐immune integration. We address this issue by constructing a machine learning driven risk‐stratification system that combines Cox regression with SHAP analysis, achieving predictive accuracy superior to traditional clinical pathology parameters. In addition, drug sensitivity analysis can identify treatment vulnerabilities with specific risks: high‐risk tumors exhibit higher sensitivity to TGF‐β inhibitors (such as LY2109761) and carmustine, while low‐risk tumors respond to metabolic modulators (EPZ004777). These findings were confirmed by tumor mutational burden (TMB) and tumor immune dysfunction and exclusion (TIDE) analyses, which revealed genomic instability and immune evasion as markers of high‐risk diseases.

## 2 Method

### 2.1 Data Collection and Data Preprocessing

The data for this study was sourced from public databases: ccRCC gene expression profiles and clinical data were obtained from the Cancer Genome Atlas (TCGA; https://portal.gdc.cancer.gov/, Version: October 2023) and Gene Expression Integrated Database (GEO; https://www.ncbi.nlm.nih.gov/geo/, Dataset GSE29609). The TCGA queue was used as the training set (containing 530 tumor samples and 72 normal samples), and the GSE29609 dataset (*n* = 39) was used as the independent validation set. The somatic mutation data were obtained from the TCGA genome atlas (version: mc3. v0.2.8) through GeneCards database (https://www.genecards.org/). Screening genes related to metabolic reprogramming, with the screening criteria being the keywords “metabolic reprogramming” and “kidney,” and the genes having ≥ 2 annotated evidences. The matrix score and immune score data calculated by ESTIMATE algorithm are from the MD Anderson Cancer Center platform (http://bioinformatics.mdanderson.org/estimate/). The TIDE score data comes from the TIDE analysis portal (http://tide.dfci.harvard.edu/). Seven hundred and three metabolic reprogramming related genes were extracted from the TCGA queue. In terms of data integration, all samples are matched through sample IDs. For multi‐omics data (transcriptome, mutation, and clinical), we used the following steps for merging and batch effect correction: (1) only retained samples with complete transcriptome, mutation, and survival information (*n* = 486); (2) using ComBat algorithm (R package sva v3.40) to perform batch effect correction on transcriptome data from different sequencing platforms; (3) standardize continuous variables (such as gene expression) using *z*‐score; (4) missing data is interpolated using the *k*‐nearest neighbor algorithm (*k* = 10). Subsequently, the TCGA and GEO datasets were merged with their corresponding clinical data for a comprehensive evaluation.

### 2.2 Differential Expression Analysis

Perform differential expression analysis using the limma package in R. Standardize the original mRNA expression data and merge gene symbols, retaining genes with an average expression level > 0.5. Comparing tumor and normal samples using the Wilcoxon rank‐sum test, with thresholds of |log FC| > 1 and FDR < 0.05, and apply the Benjamini–Hochberg method for multiple‐testing correction. Generate a heatmap of the 50 most significantly upregulated and downregulated genes using pheatmap (with standardized *z*‐values).

### 2.3 Enrichment Analysis

Perform feature enrichment analysis using the clusterProfiler package (v4.0) R. Differential genes were converted into Entrez IDs using org.Hs.eg.db, with significance thresholds set at *p* < 0.05 and FDR < 0.05, and GO (biological processes, cellular components, and molecular functions) and KEGG pathway enrichment analyses were performed. Visualize the significant enrichment results using the following methods: (1) a bar chart showing the number of genes, (2) a bubble chart showing the relationship between −log_10_(*p*‐value) and gene ratio, (3) a tree plot showing associations among functional terms, and (4) a circular plot using circlize to integrate multidimensional enrichment data.

### 2.4 MR Analysis

Two sample MR analysis was used to evaluate the causal relationship between lipid metabolism disorders and ccRCC. The genetic variation data of exposure factors (indicators related to lipid metabolism disorders) are sourced from the GWAS database (https://gwas.mrcieu.ac.uk/). The genetic data for the outcome factors (ccRCC) is sourced from the Finngen database (https://www.finngen.fi/). The main analysis method is the inverse variance weighted (IVW) method, supplemented by the weighted median method and MR Egger regression for validation to ensure the reliability of the results. The screening of instrumental variables (IVs) meets the genome‐wide significance threshold (*p* < 5 × 10^−8^) and linkage disequilibrium criteria (*r* ^2^ < 0.001). The causal effects of exposure factors were evaluated on outcomes by calculating the effect values and their significance (*p* < 0.05 is statistically significant), and visualization methods such as scatter plots were used to display correlation trends. In addition, the robustness of the results was verified through the leave one test, heterogeneity test (Cochran's *Q*), and horizontal pleiotropy analysis (MR Egger intercept test). All analyses are based on the R language (TwoSampleMR package). The genetic variation data for exposure factors (lipid metabolism disorders) is sourced from the GWAS database (ukb‐e‐272), while the genetic data for outcome factors (ccRCC) is sourced from the Finngen database (version R9, keyword KIDNEY CLEAR CELL CARCINOMA).

### 2.5 Univariate Cox Proportional Hazards Regression Analysis

Perform univariate Cox proportional hazards regression analysis using the language survival package (v3.2‐13) in R. Screening prognostic genes significantly associated with overall survival using a threshold of *p* < 0.05, and calculating the hazard ratio (HR) and 95% confidence interval for each gene. Significant prognostic genes were visualized through forest plots (*p* < 0.05).

### 2.6 Construction of Protein–Protein Interaction (PPI) Network and Hub Gene Identification

To identify core regulatory genes, a PPI network was constructed. The list of genes derived from the univariate Cox proportional hazards regression analysis was used as the input. The gene symbols were submitted to the STRING database (version 11.5) to retrieve known and predicted interactions, with a minimum required interaction score set to 0.4. The resulting PPI network was then imported into Cytoscape (version 3.9.1) for further topological analysis. Hub genes within the network were systematically identified using the cytoHubba plugin. Four distinct ranking algorithms—Degree, Maximum Neighborhood Component (MNC), Maximal Clique Centrality (MCC), and Edge Percolated Component (EPC)—were employed to evaluate the importance of each node. Subsequently, the top (e.g., 10 or 20) genes from each of the four ranking lists were selected. A Venn diagram was plotted to visualize the overlap among these gene sets. The intersecting genes common to all four methods were defined as the final robust hub genes.

### 2.7 SHAP Analysis

The feature selection adopts a three‐step method: (1) univariate Cox regression (*p* < 0.05) preliminary screening; (2) recursive feature elimination based on SHAP values for secondary screening; (3) multivariate Cox regression is used to determine the final model. Using 10‐fold cross validation for model training to prevent overfitting and select the optimal regularization parameter (*λ*). Using kernelshap (v0.3.1) and shapviz (v0.7.2) packages for SHAP analysis to explain the multivariate Cox regression model. This analysis quantifies the importance of features through the average absolute SHAP value and visualizes the results through four complementary representations. The risk stratification (high/low) is determined based on the SHAP derived risk score and validated in an independent GEO queue.

### 2.8 Principal Component Analysis (PCA)

Performing PCA using the prcomp function and feature scaling (scale = TRUE) in R (v4.1.0). Visualize the first two principal components (PC1 and PC2) using ggscatter (ggpubr v0.4.0).

### 2.9 Prognostic Analysis

Using Kaplan–Meier survival curve and Cox proportional hazards regression analysis to analyze the relationship between gene expression and survival outcomes. Perform prognostic analysis using Cox proportional hazards regression model (survival v3.2‐13). Firstly, significant clinical and molecular factors were screened through univariate analysis, and then a multivariate analysis was conducted on all significant factors. To evaluate the diagnostic and prognostic value of the identified genes, receiver operating characteristic (ROC) analysis was performed and the area under the curve (AUC) was calculated. Use a circular plot (ggplot2 v3.3.5) to display the distribution of clinical characteristics between risk groups, and use a chi‐square test for categorical variables (*p* < 0.05 is significant). All analyses were completed in R 4.1.0 using a two‐sided test.

### 2.10 Construction and Verification of Norman Diagram

Building a Norman chart based on the regplot package to visualize the prediction model. By calculating the Norman chart risk score, the accuracy of the model prediction was further evaluated using calibration curves (1 year, 3 years, and 5 years), and the consistency between the predicted survival rate and the actual observed values was verified using Bootstrap method (1000 resampling).

### 2.11 Immune Cell Infiltration Analysis

Using CIBERSORT algorithm [14] to evaluate the proportion of 22 immune cells in tumor samples, and comparing the differences in immune cell infiltration between high‐ and low‐risk groups through Wilcoxon test. Using ESTIMATE algorithm to calculate tumor microenvironment scores (stromal score, immune score, and comprehensive score), and visualize intergroup differences using violin plots. Further evaluate the activity of immune‐related functional pathways through ssGSEA analysis, quantify the enrichment scores of each functional module using GSVA algorithm, and display the differences between high‐ and low‐risk groups using box plots. For the analysis of immune subtypes, chi‐square test was used to evaluate the distribution differences of different risk groups in immune subtypes, and the subtype composition was displayed through stacked bar charts. Specifically, the CIBERSORT algorithm (version v1.03) is used for deconvolution of immune cell abundance. The core parameter settings are as follows: support vector regression uses linear kernel and nu regression types. Set the inspection frequency to 1000 times to calculate the *p*‐value; quantile normalization (QN = TRUE) is enabled by default. Only samples with CIBERSORT output *p* < 0.05 will be retained for subsequent analysis. The ESTIMATE algorithm (version 1.0.13) uses ‘Illumina’ as the platform parameter and ‘GeneSymbol’ as the gene identifier to calculate matrix score, immune score, and comprehensive score. Multiple comparisons were corrected using the Benjamini–Hochberg method to control for false discovery rate (FDR) < 0.05.

### 2.12 TMB and TIDE Analysis Methods

By integrating risk scores from TCGA data with TMB, log2 conversion was used to standardize TMB values, and the Wilcoxon test was used to compare the differences in TMB between high‐ and low‐risk groups; further determine the optimal cutoff value of TMB based on the ‘survv_cutpoint’ function of the ‘survminer’ package, and evaluate the impact of TMB and ‘TMB + risk score’ combined grouping on patient prognosis using Kaplan–Meier survival analysis. For predicting immune therapy response, the TIDE algorithm is used to calculate immune escape potential (http://tide.dfci.harvard.edu). Using default parameters (express data standardized after log2 conversion, TIDE score = functional impairment score + exclusion score), TIDE score > 0 is considered to indicate potential immune therapy resistance.

### 2.13 Drug Sensitivity Analysis

Based on GDSC2 database (https://www.cancerrxgene.org/), training a drug sensitivity prediction model, calculate the drug sensitivity score (IC_50_ value) of TCGA samples using the oncoCredit package, and standardize the data through log2 conversion. By using Wilcoxon rank‐sum test to compare the differences in drug sensitivity between high‐ and low‐risk groups, significant difference drugs were selected. The top five drugs with the highest sensitivity scores were extracted from both the low‐risk and high‐risk groups, and a grouping bar chart was drawn to show their average sensitivity scores and significance. Spearman correlation analysis was used to evaluate the correlation between risk scores and drug sensitivity for specific drugs and visualized through scatter plots.

### 2.14 Verification of Key Genes

We use diversified methods to validate the expression of key genes, independent prognostic factors, and survival status. UALCAN database (https://ualcan.path.uab.edu/) was used to analyze the expression, independent prognostic factors, and survival status of ACAT1, PC, SUCLA2, SUCLG1, and HMGCS2; Human Protein Atlas (HPA) database (https://www.proteinatlas.org/) was used to verify the differential protein expression of the core genes mentioned above between normal tissues and ccRCC tissues. The human renal cell carcinoma cell lines (786‐O and ACHN) and the human renal proximal tubular cell line (HK‐2) used for validation of core gene mRNA expression experiments were obtained from the American Type Culture Bank (ATCC, USA). 786‐O and ACHN cells were cultured in RPMI‐1640 medium supplemented with 10% fetal bovine serum (FBS), while HK‐2 cells were maintained in Dulbecco modified eagle medium (DMEM) containing 10% FBS. Both cell lines were incubated at 37°C in a humidified atmosphere of 5% CO_2_. Cell lines were routinely authenticated and tested for mycoplasma contamination to ensure experimental reliability. For quantitative real‐time polymerase chain reaction (qRT‐PCR) analysis, commercial RNA extraction kits were used to extract total RNA from cultured cells. Eliminate genomic DNA and follow the manufacturer's PrimeScript instructions for synthesizing cDNA using RT kit (Takara, Beijing, China). Prepare the qRT‐PCR reaction mixture according to the kit protocol and amplify it using the Applied Biosystems 7500 real‐time fluorescence quantitative PCR system (Thermo Fisher Scientific, Shanghai, China). Normalize the relative mRNA expression level of the target gene to GAPDH (or β‐actin) as an internal control and calculate using the 2^−ΔΔCt^ method. The primer sequences of five core genes and internal references are shown in Table S1. Total protein was extracted from HK‐2, 786‐O, and ACHN cells using RIPA buffer. After quantification by BCA assay, equal amounts of protein were separated by SDS‐PAGE and transferred to PVDF membranes. Membranes were blocked with 5% nonfat milk and then incubated with specific primary antibodies (Table S2) overnight at 4°C. Following incubation with HRP‐conjugated secondary antibodies, protein bands were visualized using an ECL reagent. β‐Actin/GAPDH served as a loading control. Data are presented as the mean ± standard deviation (SD) from at least three independent experiments. Statistical analyses were performed using GraphPad Prism software (version 8.0, USA). Statistical significance was assessed using Student's *t*‐test. A *p*‐value less than 0.05 is considered statistically significant.

### 2.15 Cell Culture, Transfection, and Functional Assays

For functional studies, SUCLA2 overexpression was achieved by transfecting cells with a pcDNA3.1‐SUCLA2 plasmid, while knockdown was performed using small interfering RNA targeting SUCLA2 (si‐SUCLA2: 5′‐GGAUCAAGUUCGACGACUATT‐3′). Both constructs were synthesized by GenePharma (Shanghai, China). Transfections were carried out using Lipofectamine 3000 (Invitrogen, USA) according to the manufacturer's instructions when cells reached approximately 50% confluence. After 48 h of transfection, cells were harvested for subsequent experiments. Cell proliferation was assessed using the Cell Counting Kit‐8 (CCK‐8, Dojindo, Japan) at 0, 12, 24, 48, and 72 h post‐seeding. Migration ability was evaluated by wound healing assays, and scratch closure was monitored and quantified after 24 h. For Transwell assays, 2 × 10^4^ cells were seeded into the upper chambers with or without Matrigel coating to evaluate migration and invasion, respectively. After 24 h of incubation, cells that migrated or invaded to the lower surface were fixed, stained with crystal violet, and counted under a microscope.

### 2.16 Statistical Analysis

All experiments were performed from at least three independent biological replicates, and data are presented as mean ± SD. Statistical analyses were conducted using GraphPad Prism 8.0 (USA). Comparisons between two groups were evaluated using unpaired two‐tailed Student's *t*‐test. For multiple group comparisons, one‐way or two‐way analysis of variance (ANOVA) was applied, followed by Tukey's or Dunnett's post hoc test for pairwise comparisons. A *p*‐value less than 0.05 was considered statistically significant (*p < 0.05, **p < 0.01, and ***p < 0.001).

## 3 Results

### 3.1 Differential Expression and Enrichment Analysis

**FIGURE 1:**
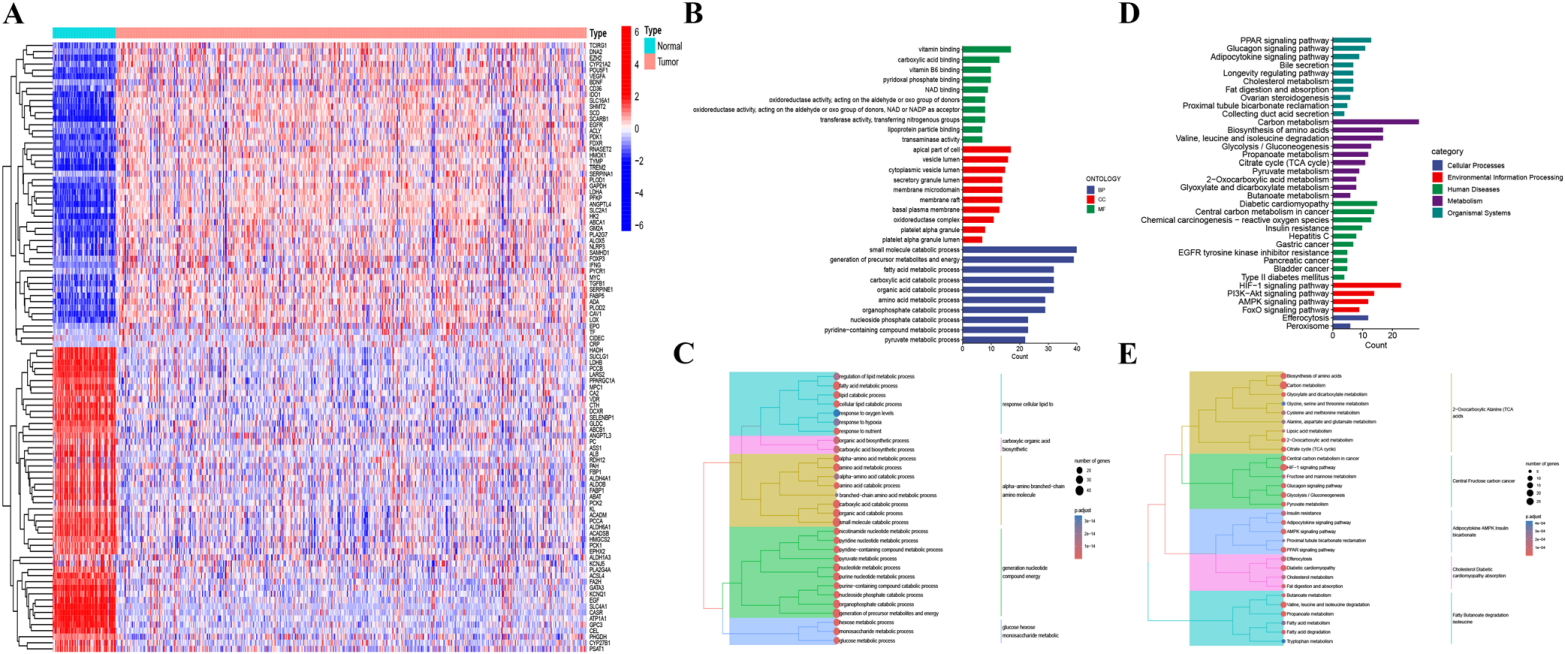
Metabolic reprogramming analysis in tumor samples. (A) Heatmap showing the expression patterns of key metabolic genes in normal versus tumor tissues. Rows represent genes, columns represent samples, and colors indicate *Z*‐score‐normalized expression levels (red: high; blue: low). (B) GO enrichment analysis; bar chart of top enriched molecular functions and cellular components. (C) Tree diagram of enriched biological processes related to lipid metabolism and amino acid metabolism. (D) KEGG pathway enrichment analysis, with bar charts highlighting metabolic pathways and disease‐related pathways. (E) The tree diagram of enriched biological processes displays enrichment pathways.

To identify differentially expressed genes (DEGs) related to metabolic reprogramming that play a key role in ccRCC, the “limma” package was used to compare gene expression profiles between cancerous and normal tissues (Figure 1A). The functional enrichment analysis revealed significant involvement of genes in key metabolic pathways and biological processes. Gene Ontology (GO) terms highlighted the regulation of lipid metabolic processes, fatty acid metabolism, and cellular lipid catabolism, alongside responses to oxygen levels and hypoxia. Notably, amino acid metabolic processes, particularly alpha‐amino acid and branched‐chain amino acid metabolism, were prominently enriched, with significant *p*‐values (p.adjust < 0.05). Additionally, carbohydrate metabolism, including glucose, hexose, and monosaccharide metabolic processes, was strongly associated with the gene set (Figure 1B).

KEGG pathway analysis further underscored these findings, identifying central carbon metabolism, the citrate cycle (TCA cycle), and glycolysis/gluconeogenesis as critical pathways. Amino acid biosynthesis pathways, such as glycine, serine, threonine, and cysteine metabolism, were also significantly enriched. Pathways related to energy homeostasis, including the AMPK signaling pathway and insulin resistance, were implicated, along with lipid metabolism pathways like fatty acid degradation and cholesterol metabolism. The HIF‐1 signaling pathway, linked to hypoxia response, further aligned with the GO results. These findings collectively suggest a coordinated regulation of metabolic processes, emphasizing roles in nutrient response, energy generation, and hypoxia adaptation (Figure 1D).

The tree diagram further visualizes the above results (Figure 1C,E). The high statistical significance (p.adjust < 0.05) and the convergence of GO and KEGG results reinforce the biological relevance of these pathways in the studied context.

### 3.2 Causal Analysis of Exposure Factors and Outcome Factors

**FIGURE 2:**
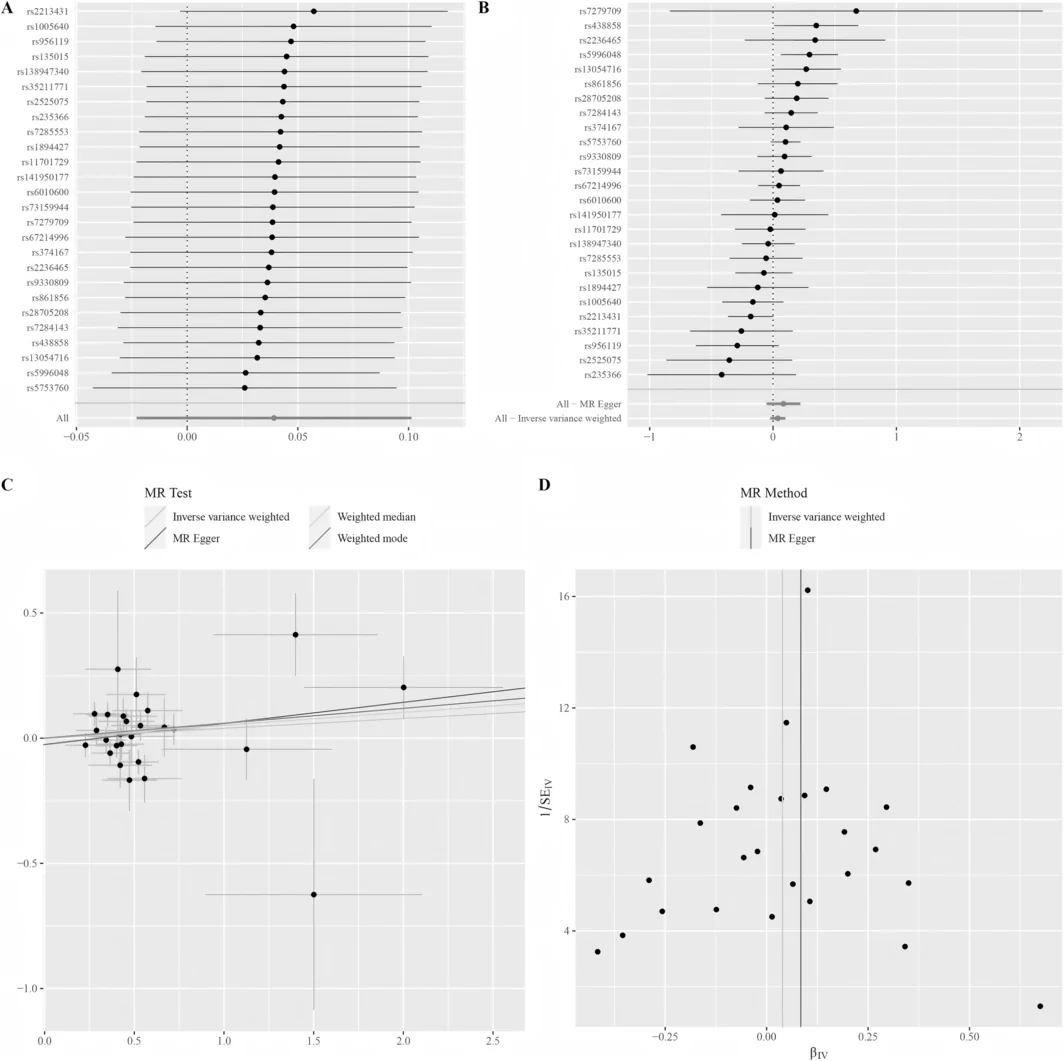
The causal relationship between lipid metabolism disorders and ccRCC. (A) Conduct a sensitivity analysis using the leave one method to determine if there is a single SNP that has a significant impact on the association. (B) Evaluate the causal effects of each SNP estimation exposure on the results and represent them in a forest plot. (C) Plot the impact of SNPs on outcomes and their effects on exposure. The slope of the line represents causal association, and each method has a different line. (D) The funnel plot is used to evaluate heterogeneity.

Based on the results of the enrichment analysis mentioned above, we consider lipid metabolism disorders as exposure factors and ccRCC as outcome factors. Based on the exposure factor and outcome factor datasets, we conducted a MR study to explore the causal relationship between the two. The selection criteria for IVs are genome‐wide significance (*p* < 5 × 10^−8^) and low linkage disequilibrium (*r* ^2^ < 0.001). The sensitivity analysis explained that after removing each SNP, the overall error line did not change significantly, meaning that all error lines were basically on the right side of 0, indicating that the causal relationship results were reliable (Figure 2A). The SNP forest plot revealed using the Wald ratio method that an MR effect greater than 0 confirms that lipid metabolism disorder is a risk factor for ccRCC (Figure 2B). The scatter plot shows a positive correlation (slope > 0) between lipid metabolism disorder and ccRCC (Figure 2C). The funnel plot is mainly used to evaluate potential biases in MR analysis. The distribution of SNPs in the figure is roughly symmetrical, without obvious skewness or clustering, indicating a low possibility of horizontal pleiotropy or selection bias. The MR Egger and IVW methods have overall consistent effect estimates for different SNPs, providing solid support for the reliability of causal effect analysis (Figure 2D; Table S3). It should be emphasized that these findings provide evidence for the association between genetic prediction of lipid metabolism disorders and ccRCC risk, but cannot be explained as a definitive causal mechanism.

### 3.3 Screening of Prognostic Genes and SHAP Analysis

**FIGURE 3:**
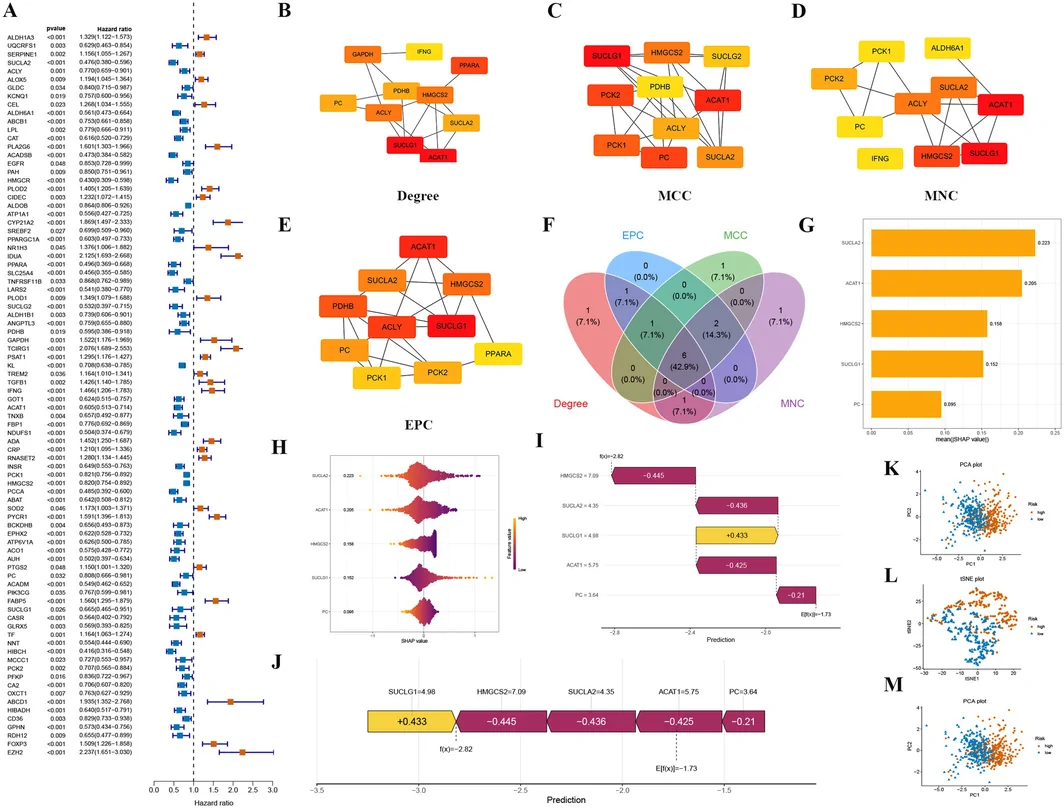
Identification of metabolic reprogramming core genes. (A) Forest map displays metabolic reprogramming genes associated with patient survival. Hub gene identification within the PPI network. (B‐E) Topological ranking of nodes in the refined PPI network using four Cytoscape algorithms: (B) Degree, (C) MCC, (D) MNC, and (E) EPC. Red nodes indicate genes with the highest centrality scores in each algorithm, while yellow nodes represent lower‐ranked candidates. (F) The intersection of the top‐ranked genes from all four algorithms yielded the final set of core hub genes. (G) The bar chart of top metabolic genes ranked by average absolute SHAP values reflects their contribution to the prediction model. (H) The bee colony plot displays the distribution of SHAP values for key genes, colored by eigenvalues (low/high expression). (I) Waterfall diagram illustrating the contribution of a single feature to sample risk prediction. (J) Striving to visualize the directional impact of top‐level genes on model predictions. (K–M) Three different methods for stratifying samples.

Following the identification of 145 differentially expressed metabolic reprogramming genes, univariate Cox regression analysis was performed, which identified 86 genes with significant prognostic value (Figure 3A). To further refine this gene set and identify core regulatory targets, we constructed a PPI network. Hub genes within this network were systematically identified using four topological algorithms (Degree, MCC, MNC, and EPC) in Cytoscape (Figure 3B–E). The intersection of the top‐ranked genes from these four methods yielded six robust core genes for subsequent analysis (Figure 3F). These six core genes were fed into a SHAP‐based prognostic modeling pipeline to construct a multivariate Cox model and interpret feature importance. The SHAP analysis revealed the key contributions of these genes to the ccRCC risk prediction model. A bar plot, ranked by the mean absolute SHAP values, identified SUCLA2 (0.223), ACAT1 (0.205), HMGCS2 (0.158), SUCLG1 (0.152), and PC (0.095) as the most influential biomarkers (Figure 3G). A beeswarm plot further illustrates the impact of each feature across the entire cohort. Except for the increased expression of SUCLG1 and SHAP in the five core targets, the other four genes are negatively correlated with SHAP values (Figure 3H). To elucidate how these genes collectively shape patient‐specific predictions, a force plot visualized the transition from the baseline model output (*E*[*f*(*X*)] = −1.73) to the individual prediction for a representative sample (*f*(*x*) = 2.82). This analysis highlighted SUCLA2, ACAT1, PC, SUCLG1, and HMGCS2 as the core drivers of the prognostic signature (Figure 3I,J). Subsequently, we employed dimensionality reduction techniques (PCA, UMAP, and t‐SNE) to evaluate the model's discriminatory power. The results confirmed that the expression profiles of the model genes could effectively separate samples from high‐ and low‐risk groups (Figure 3K–M).

### 3.4 Survival and Independent Prognostic Analysis

**FIGURE 4:**
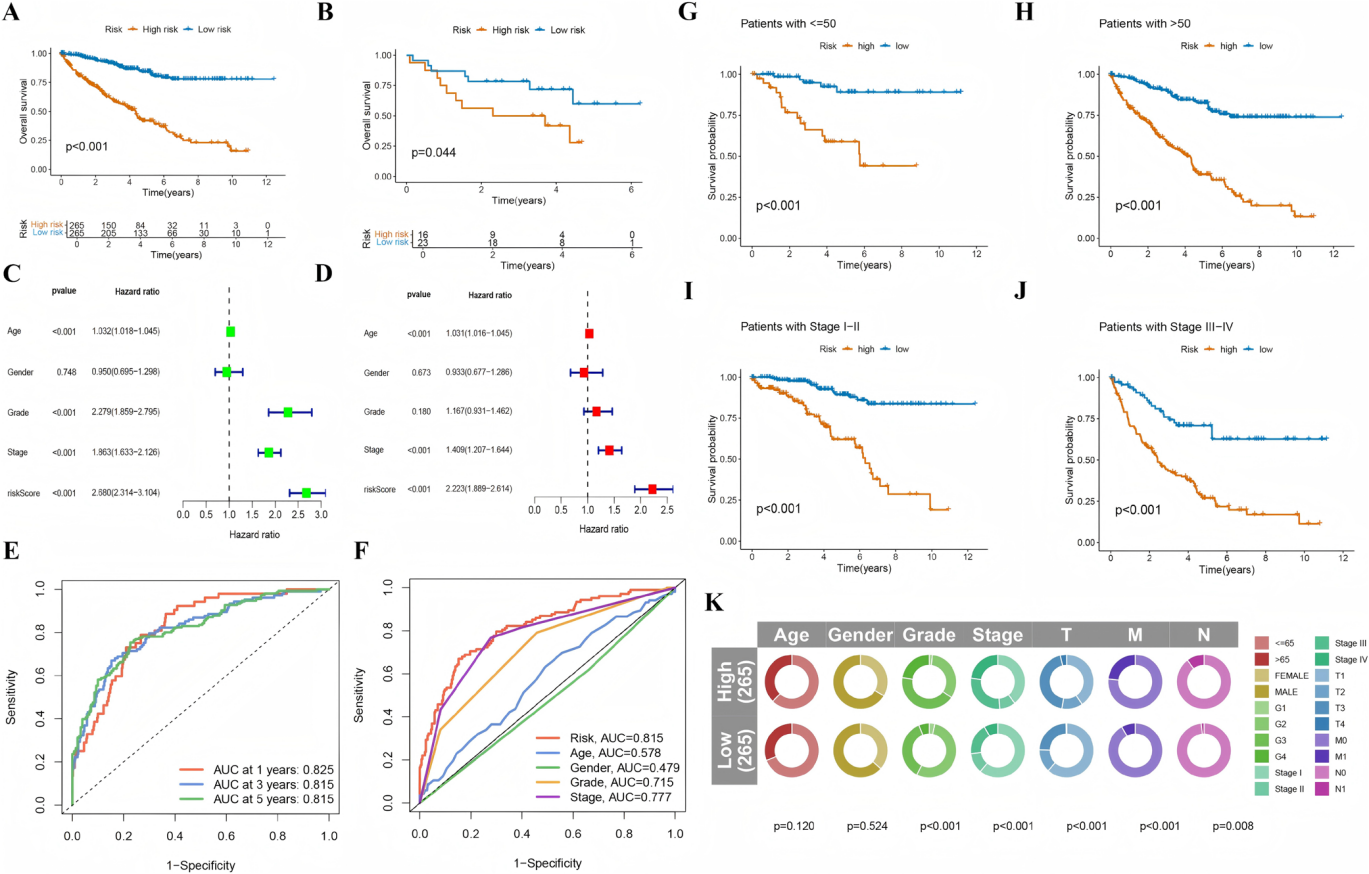
Validation of independent prognostic factors in ccRCC. (A) Survival curves of high‐risk (*n* = 243) and low‐risk (*n* = 243) groups in the TCGA cohort (log rank test, *p* < 0.001). (B) Survival curves in GEO queue (*n* = 39, log rank test, *p* = 0.012). (C and D) Forest plot of independent prognostic factors in TCGA. (E) ROC curve used to evaluate the predictive efficiency of survival prognostic features. (F) ROC curve used to evaluate the predictive efficiency of prognostic features in the model. (G and H) Kaplan–Meier survival curves for age groups. (I and J) Kaplan–Meier survival curves for stage groups. (K) Correlation circle diagram of clinical traits between two risk groups (chi‐square test, *p* < 0.05).

Survival analysis conducted in the TCGA (training cohort) and GEO (validation cohort) databases showed that the low‐risk and high‐risk groups had significantly better prognoses (Figure 4A,B, *p* < 0.05). Multivariate Cox regression identified age, tumor stage, and risk score as independent prognostic factors for ccRCC (Figure 4C,D, *p* < 0.05). The model exhibits strong diagnostic performance, with time‐dependent ROC curves showing AUC values of 0.825 (1 year), 0.815 (3 years), and 0.815 (5 years) (Figure 4E). It is worth noting that riskScore achieved the highest prediction accuracy (AUC = 0.815, Figure 4F), surpassing conventional clinical parameters. The subsequent stratified analysis confirmed the prognostic value of riskScore in all age groups and tumor stages (Figure 4G–J, *p* < 0.05). Significant correlations were observed between risk stratification and key clinical features such as histological grading, TNM staging, and transition status (Figure 4K, *p* < 0.05), validating the clinical relevance of our risk classification system.

### 3.5 Construction of Norman Diagram and Analysis of Immune Infiltration

**FIGURE 5:**
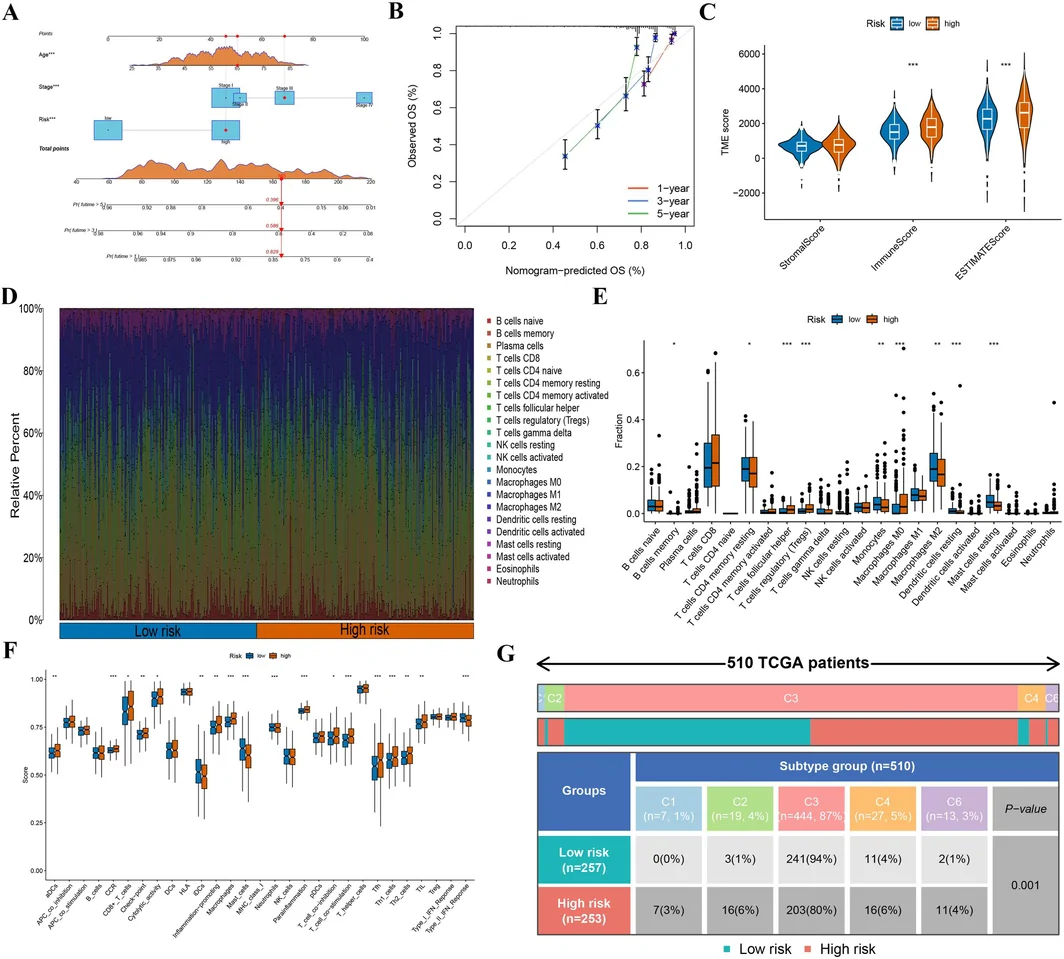
Construction of Norman diagram and immune infiltration. (A) A column chart of a risk model used to predict the survival probability of ccRCC patients. The integer points projected onto the bottom scale represent the likelihood of 1‐year, 3‐year, and 5‐year OS. (B) Calibration chart of column charts predicting survival for 1, 3, and 5 years. (C) Immune, stromal, and ESTIMATE scores with the low‐ and high‐risk groups (*n* = 265). (D and E) Boxplots showed abundance of 22 infiltrating immune cell types. (F) Immune function differential analysis. (G) Differences in immune typing between two risk groups.

We included independent prognostic factors in the column chart to predict the survival probability of individual patients (Figure 5A). The calibration curve shows a strong agreement between the predicted and observed results of 1‐year, 3‐year, and 5‐year survival periods (Figure 5B). Tumor microenvironment analysis showed that the ESTIMATE and immune scores (both *p* < 0.01) of the high‐risk group were significantly higher, but the stromal scores were comparable, indicating lower tumor purity (Figure 5C). A comprehensive immune analysis revealed different immunological landscapes: Low‐risk tumors showed enrichment of resting CD4^+^ memory T cells (*p* < 0.01) [15], monocytes (*p* < 0.05) [16], and resting dendritic cells (*p* < 0.05) [17], indicating preservation of immune surveillance, while paradoxically, increased M0 (*p* < 0.01) and M2 macrophages (*p* < 0.05) may indicate a plastic bone marrow state [18]. In contrast, high‐risk tumors exhibit significant accumulation of immunosuppressive subgroups, including Tfh (*p* < 0.001) and Tregs (*p* < 0.01), creating an immune evasion niche—findings consistent with the metabolic drivers identified by our SHAP (HMGCS2), which may promote Treg recruitment through lactate‐mediated immunosuppression [19] (Figure 5D,E). Further functional analysis showed that the immune regulatory components of low‐risk tumors were enhanced, including iDC (*p* < 0.01), mast cells (*p* < 0.001), neutrophils (*p* < 0.001), and IFN‐γ response (*p* < 0.001), in sharp contrast to the immunosuppressive features of the high‐risk group (elevated checkpoint molecules, T cell coinhibition, and impaired cell lysis activity) (Figure 5F). The immunological subtypes of 510 TCGA patients showed significant distribution differences (*p* = 0.001), with low‐risk tumors mainly classified as immune heat C3 subtype (94%). The strong association between risk‐stratification and immune subtypes validated the ability of our model to capture biologically different tumor microenvironments (Figure 5G).

### 3.6 Tumor Mutation Analysis

**FIGURE 6:**
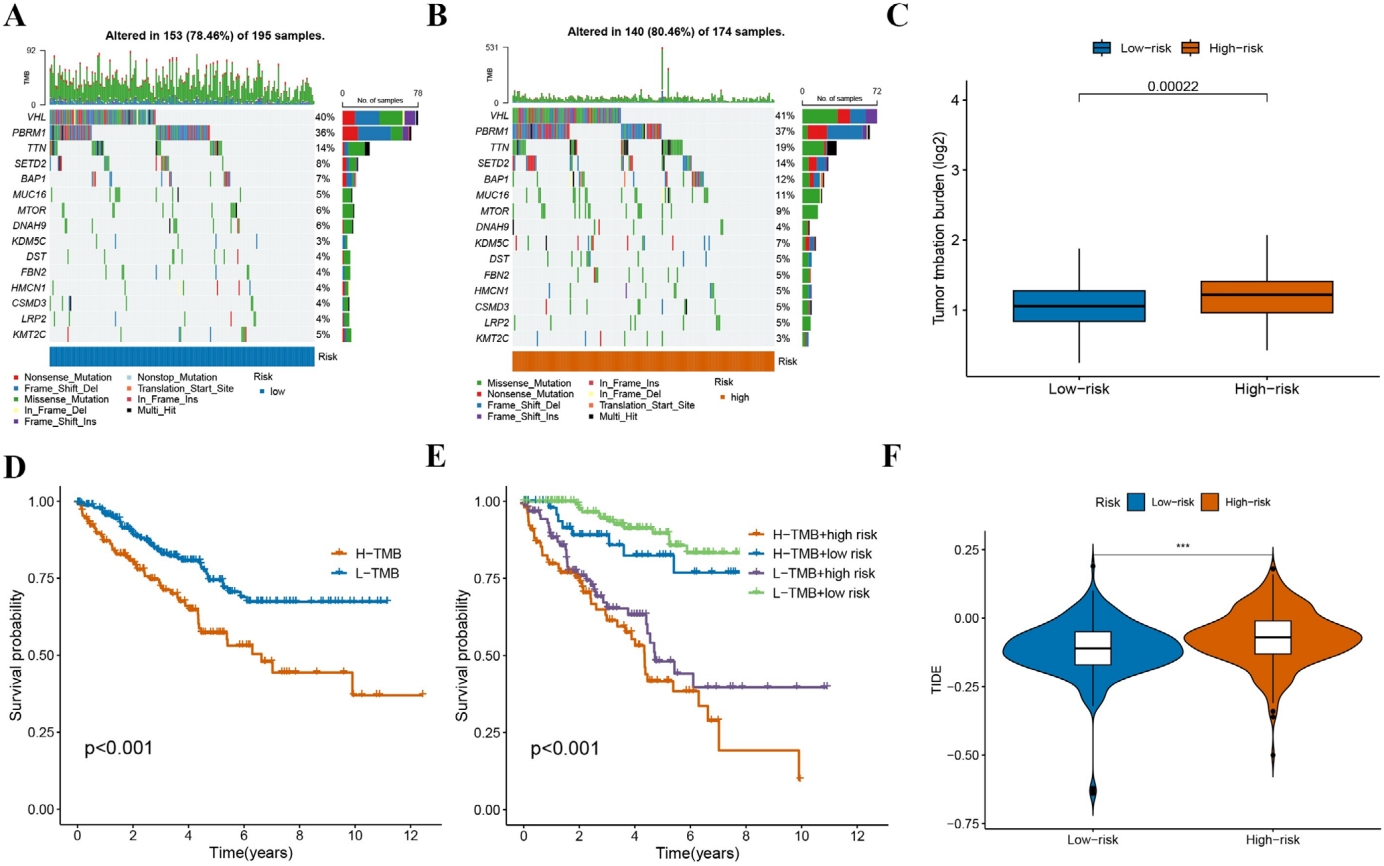
Tumor mutation and immunotherapy analysis. Gene mutation in the (A) low‐risk group and (B) high‐risk group. (C) Difference of TMB between high‐ and low‐risk groups (*n* = 265). (D) Survival curves of high and low TMB groups. (E) Survival curve of TMB grouping combined with risk groupings. (F) TIDE scores with the low‐ and high‐risk groups.

Genomic analysis revealed different mutation patterns among risk‐stratified ccRCC groups. Although VHL and PBRM1 mutations were equally common in both groups without significant differences, other key driver genes (including TTN, SETD2, and BAP1) showed significant differences (Figure 6A,B). It is worth noting that compared with low‐risk cases, high‐risk tumors exhibit significantly increased TMB and distinct mutation characteristics (TMB, *p* < 0.05, Figure 6C). Survival analysis showed that the prognosis of the low TMB/low‐risk group was better than that of high TMB/high‐risk patients with poor prognosis (Figure 6D,E), indicating the synergistic effect of genomic instability and immune microenvironment on clinical outcomes. The high‐risk group showed greater potential for immune escape, as evidenced by increased TIDE scores (Figure 6F), indicating a reduced likelihood of response to immune checkpoint inhibitors [20]. These findings provide mechanistic insights into the interaction between somatic mutations and immune escape, where high‐risk tumors combine genomic instability with immunosuppressive features to drive aggressive behavior. The intergroup differential mutation landscape and TMB spectrum may provide information for personalized treatment strategies, and high TMB/high‐risk cases may require a combination approach targeting genomic instability and immune resistance pathways.

### 3.7 Potential Drug Screening

**FIGURE 7:**
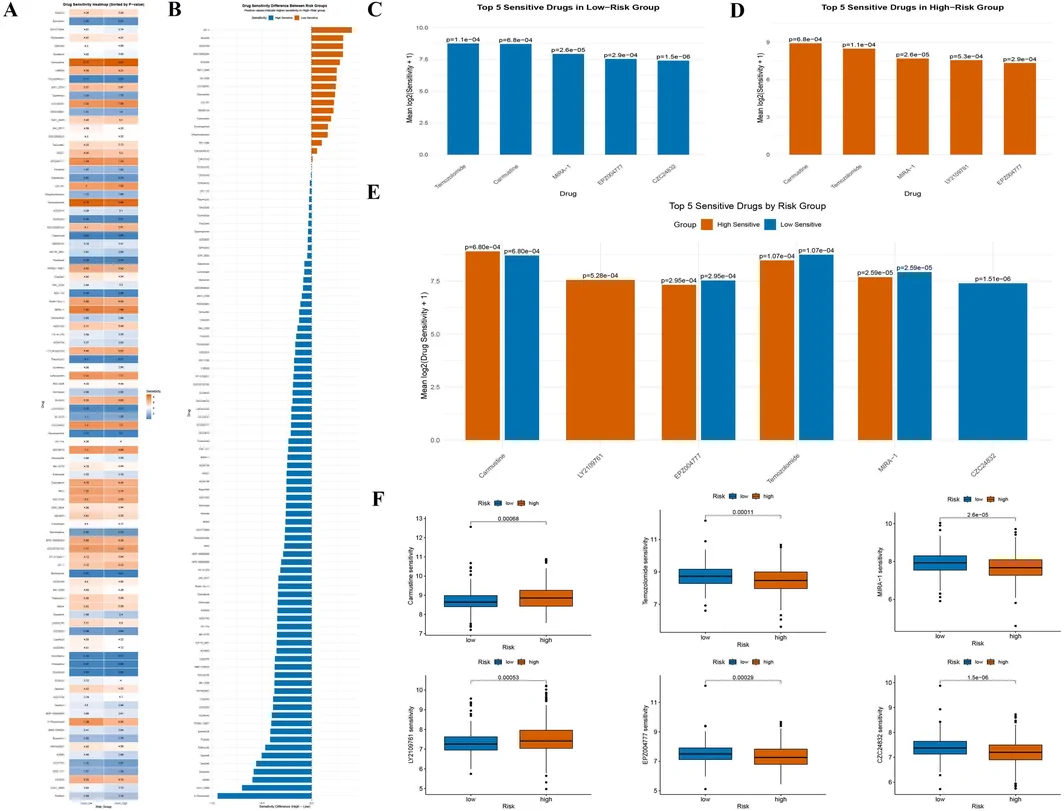
Correlation between drug sensitivity and risk groups. (A) Heat map of drug sensitivity between two risk groups. (B) Waterfall plot of drug sensitivity between two risk groups. (C) The top five most sensitive drugs in the low‐risk group (*n* = 265). (D) The top five most sensitive drugs in the high‐risk group (*n* = 265). (E) Distribution of the top five most sensitive drugs in the two risk groups. (F) Differences in sensitivity of six drugs between two risk groups. **p < 0.01; ***p < 0.001; ****p < 0.0001.

**FIGURE 8:**
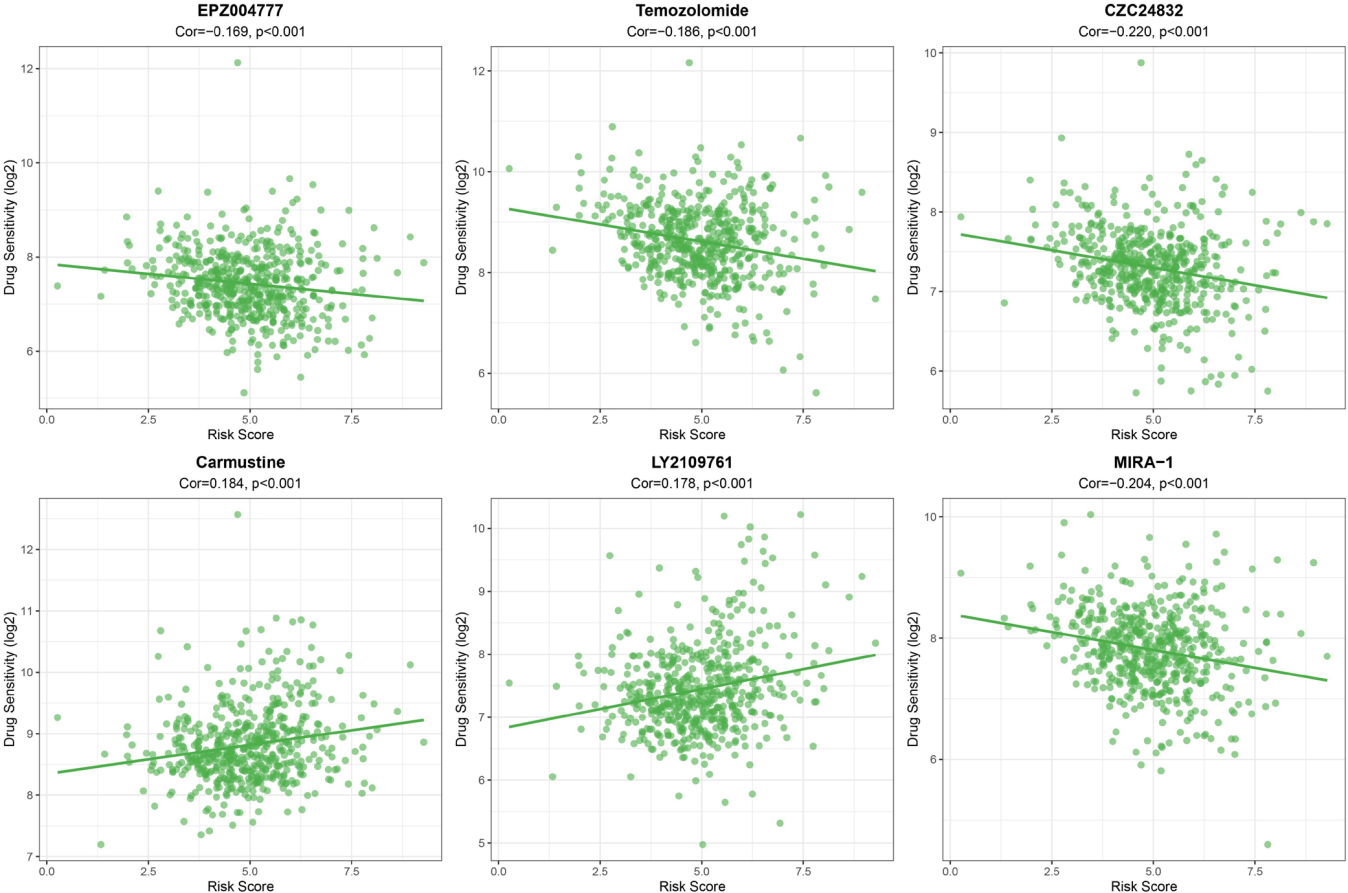
The correlation between sensitivity to six drugs and risk scores.

Using GDSC database (https://www.cancerrxgene.org/), we systematically screened drug sensitivity patterns in ccRCC. Visualize potential therapeutic drugs through sensitivity heatmaps and waterfall plots (Figure 7A,B). In order to identify the most promising candidate drugs, we selected the top five sensitive drugs for each risk group: temozolomide, carmustine, MIRA‐1, EPZ004777, and CZC24832 showed the highest sensitivity in the low‐risk group (Figure 7C), while carmustine, temozolomide, MIRA‐1, LY2109761, and EPZ004777 were the most effective in the high‐risk group (Figure 7D). Comparative analysis shows that four drugs (carmustine, temozolomide, MIRA‐1, and EPZ004777) have cross‐risk group efficacy (Figure 7E). These six drugs showed statistically significant differences between subgroups (all *p* < 0.05). Further correlation analysis confirmed that LY2109761 (Cor = 0.184, *p* < 0.001) and carmustine (Cor = 0.178, *p* < 0.001) are particularly promising for high‐risk patients (Figure 8), indicating their potential as risk‐stratification treatment options.

### 3.8 Expression Analysis and Prognostic Value of Five Core Genes in Relation to Independent Risk Factors

**FIGURE 9:**
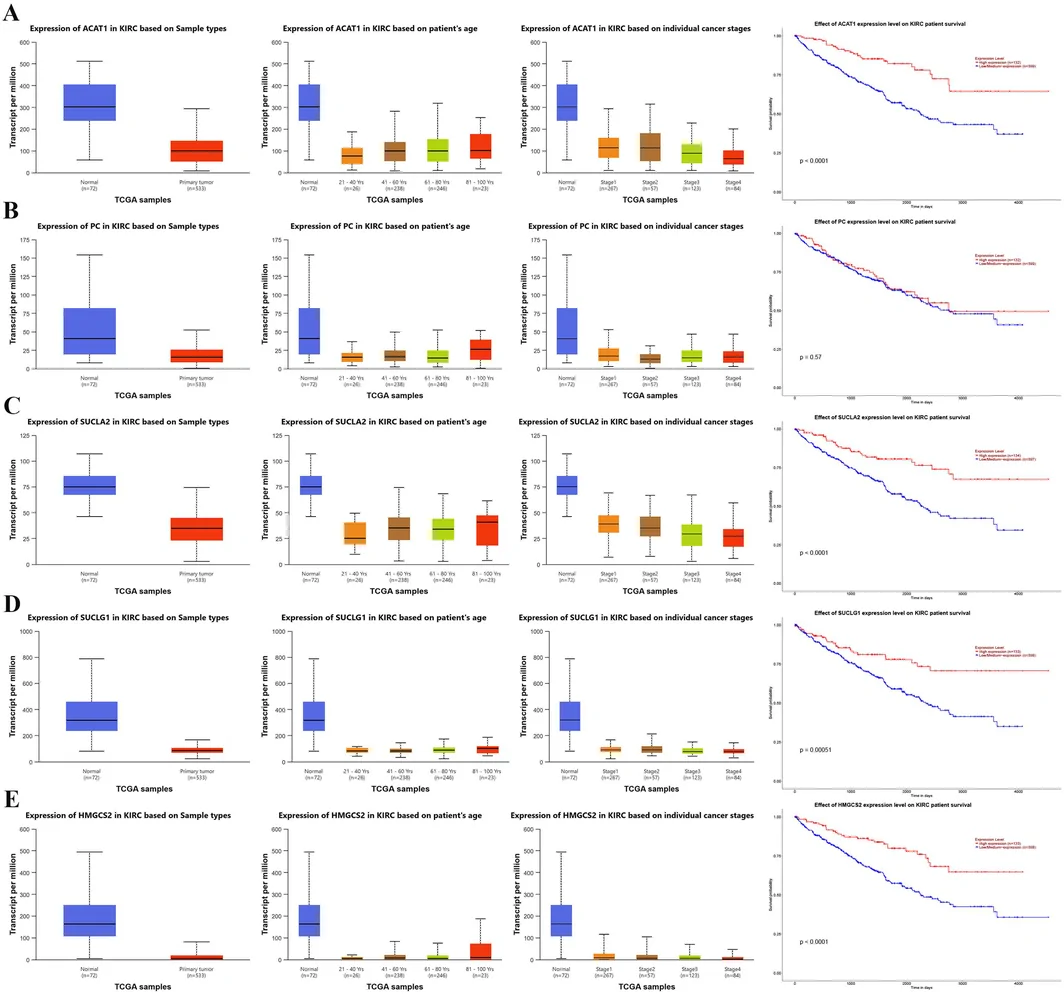
Expression patterns and prognostic significance of the five core genes in relation to age and tumor stage. (A) ACAT1 expression is upregulated in tumor tissues and correlates with poor prognosis. Its expression increases with advanced age and higher tumor stage. Expression of (B) PC, (C) SUCLA2, (D) SUCLG1, and (E) HMGCS2 is downregulated in tumor tissues and correlates with favorable prognosis. Their expression decreases with advanced age and higher tumor stage. Data were analyzed using the UALCAN database. All compared to the normal group; ***p < 0.001; ****p < 0.0001.

**FIGURE 10:**
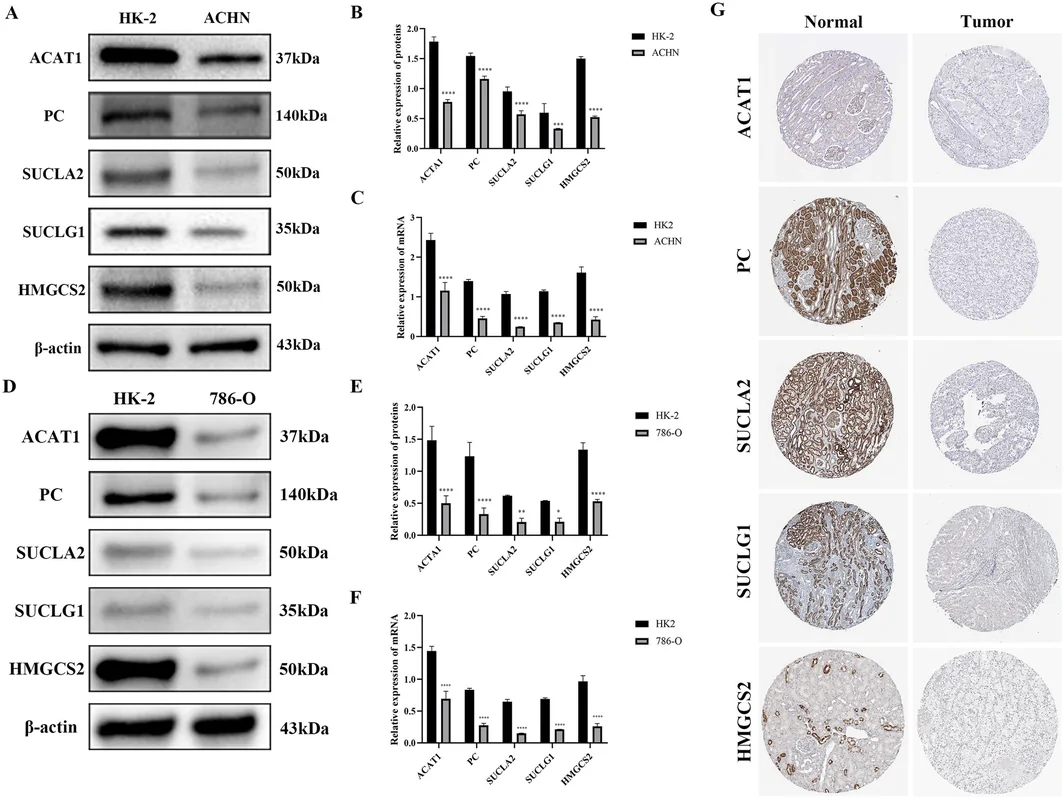
Validation of protein and mRNA expression levels of the five core genes. Immunohistochemical images from the Human Protein Atlas (HPA) database, quantitative PCR, and western blot results confirm the protein and transcriptional expression patterns of (A–C) protein expression levels of the five core genes in the ACHN cell line. (B–F) mRNA expression levels of the five core genes in the 786‐O cell line. (G) Immunohistochemical images of the five core genes. *p < 0.05; **p < 0.01; ***p < 0.001; ****p < 0.0001.

Using online databases, we investigated the expression patterns of five core genes (SUCLA2, ACAT1, PC, SUCLG1 and HMGCS2) and their relationship with two established independent prognostic factors (age and tumor stage). Analysis through the UALCAN database showed that five core genes showed low expression in tumor tissues, and their high expression predicted better prognosis. It is worth noting that their expression gradually decreases with increasing age and tumor stage (Figure 9A–E). In addition, our RT‐PCR and western blot results further support these findings, with ACHN (Figure 10A–C) and 786‐O (Figure 10D–F) cell lines showing consistent expression patterns at the transcriptional and protein level. Subsequently, validation using the HPA database confirmed these trends at the tissue protein level for all five genes (Figure 10G).

### 3.9 SUCLA2 Suppresses ccRCC Malignant Phenotypes In Vitro

**FIGURE 11:**
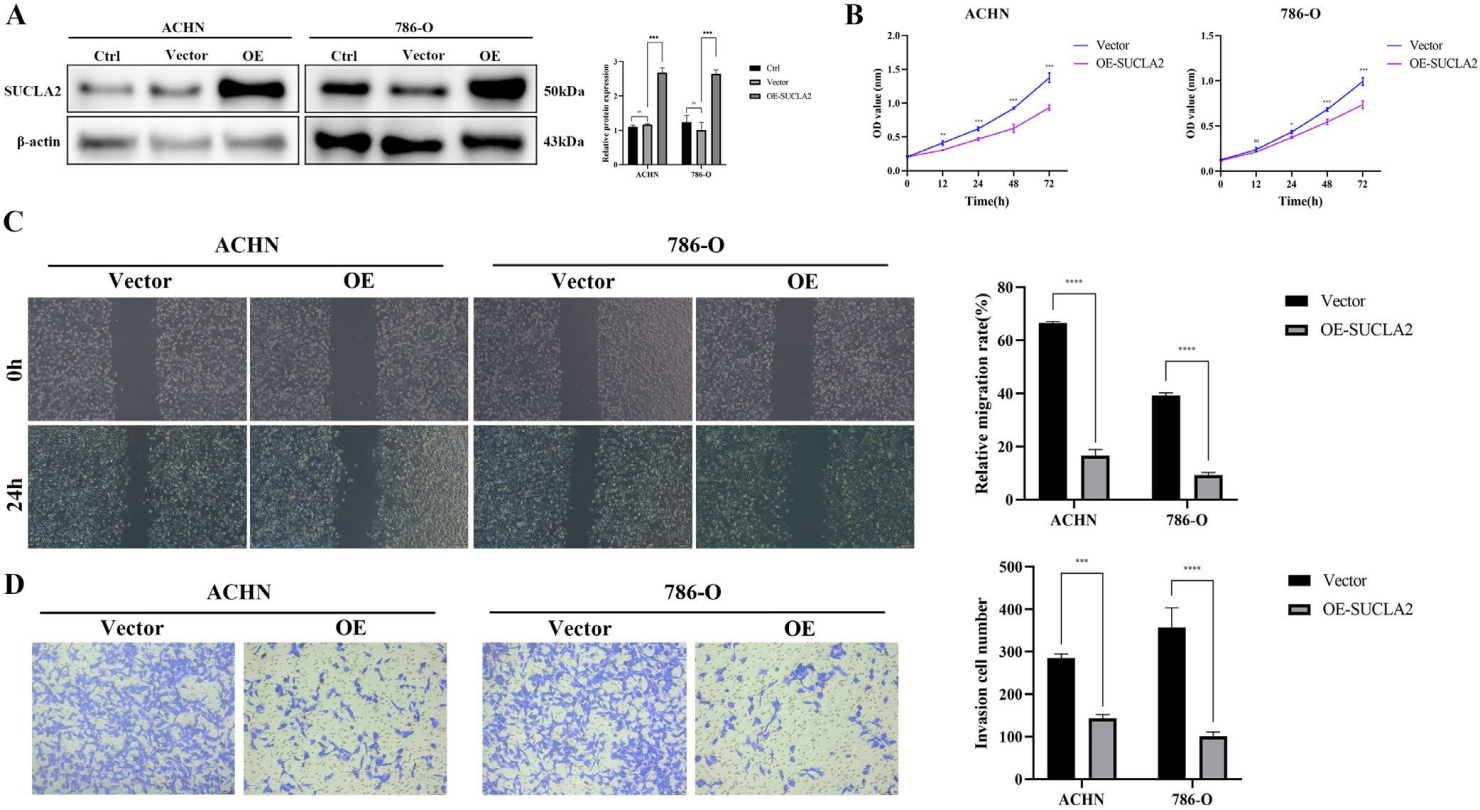
Overexpression of SUCLA2 suppresses malignant phenotypes in ccRCC cells. (A) Western blot analyses confirming SUCLA2 protein expression levels in 786‐O and ACHN cells transfected with OE‐SUCLA2 or empty vector. (B) Cell proliferation was assessed by CCK‐8 assays at the indicated time points in 786‐O and ACHN cells following SUCLA2 overexpression. (C) Wound healing assays evaluating the migratory capacity of SUCLA2‐overexpressing cells. (D) Matrigel invasion assays were performed to assess the effects of SUCLA2 overexpression on cell invasiveness. All experiments were performed in triplicate. *n* = 3, Student's *t*‐test. Data are presented as mean ± SD. *p < 0.05, **p < 0.01, and ***p < 0.001 compared to the vector group.

**FIGURE 12:**
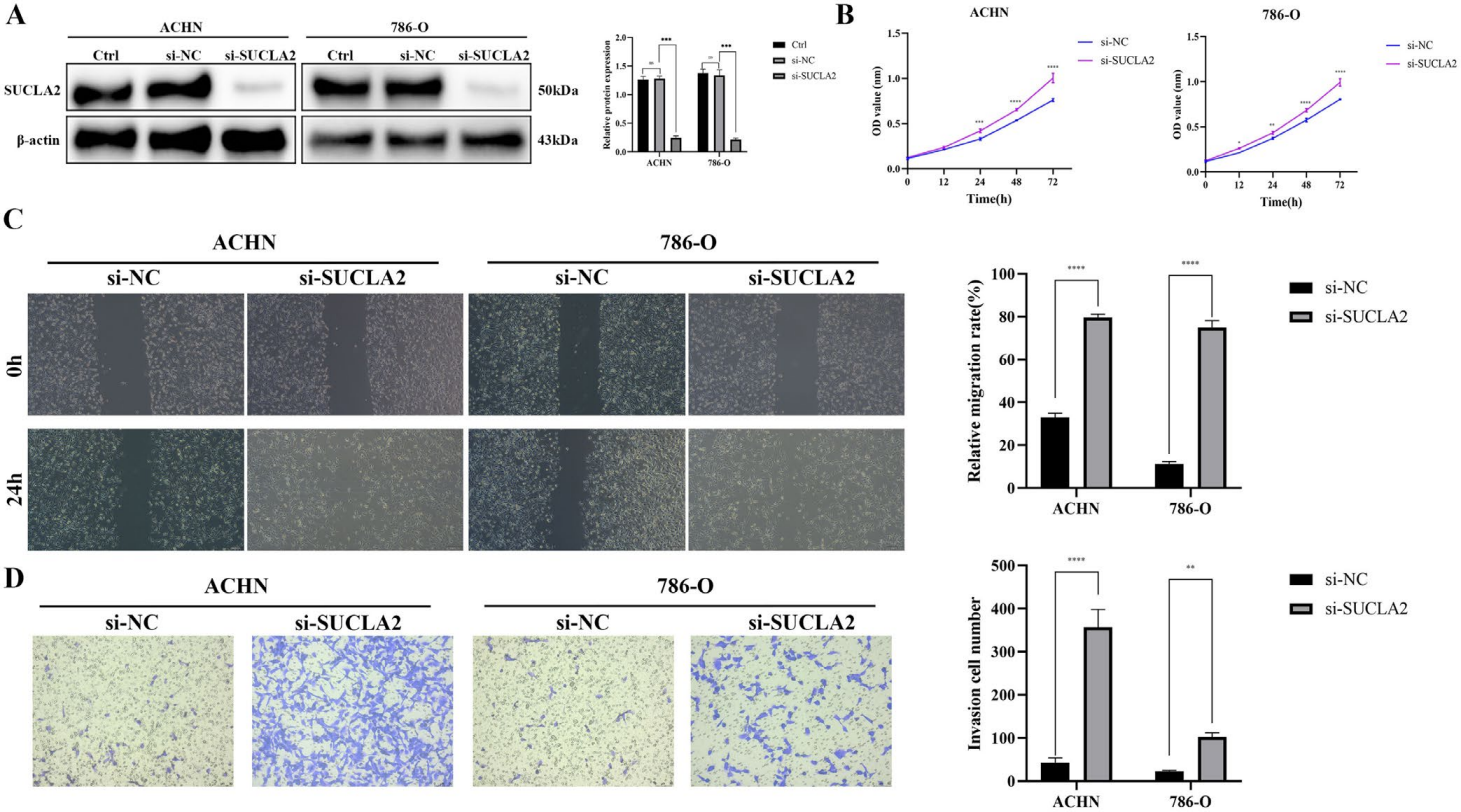
Knockdown of SUCLA2 promotes malignant phenotypes in ccRCC cells. (A) Western blot analyses confirming SUCLA2 protein expression levels in 786‐O and ACHN cells transfected with si‐SUCLA2 or negative control siRNA (si‐NC). (B) CCK‐8 assays showing the proliferative capacity of 786‐O and ACHN cells after SUCLA2 knockdown at the indicated time points. (C) Wound healing assays evaluating the migratory capacity of SUCLA2‐silenced cells. (D) Matrigel invasion assays were performed to assess the effects of SUCLA2 knockdown on cell invasiveness. All experiments were performed in triplicate. *n* = 3, Student's *t*‐test. Data are presented as mean ± SD. *p < 0.05, **p < 0.01, ***p < 0.001, and ****p < 0.0001 compared to the si‐NC group.

Many studies have demonstrated the predictive role of HMGCS2 [21], ACAT1 [22], SUCLG1 [23], and PC [24] in ccRCC. Given the lack of functional studies on SUCLA2 in ccRCC, we first validated the efficiency of overexpression and knockdown in 786‐O and ACHN cells. Western blot analyses confirmed that transfection with OE‐SUCLA2 significantly increased SUCLA2 protein levels (Figure 11A), while treatment with si‐SUCLA2 effectively downregulated its expression compared to negative controls (Figure 12A). We then investigated the biological role of SUCLA2 through gain‐ and loss‐of‐function experiments. CCK‐8 assays showed that overexpression of SUCLA2 significantly reduced cell proliferation in both 786‐O and ACHN cells (Figure 11B). Conversely, knockdown of SUCLA2 enhanced proliferative capacity (Figure 12B). Wound healing assays demonstrated that SUCLA2 overexpression impaired cell migration (Figure 11C), while SUCLA2 silencing accelerated wound closure (Figure 12C). Consistently, Transwell migration and invasion assays revealed that SUCLA2 overexpression suppressed both migratory and invasive abilities (Figure 11D), whereas SUCLA2 knockdown promoted these malignant behaviors (Figure 12D). Collectively, these findings indicate that SUCLA2 functions as a tumor suppressor in ccRCC by inhibiting proliferation, migration, and invasion.

## 4 Discussion

This multi‐omics study systematically depicted the metabolic reprogramming characteristics of ccRCC and established a robust prognostic model by integrating bioinformatics methods. The research results reveal three fundamental aspects of ccRCC biology: (1) the core role of metabolic disorders in tumor progression; (2) the complex interaction between metabolic changes and immune microenvironment remodeling; (3) the clinical value of risk stratification in individualized treatment decision‐making.

Differential expression and enrichment analysis identified lipid metabolism, amino acid metabolism, and carbohydrate metabolism as key pathways in the pathogenesis of ccRCC. The consistency between GO and KEGG results strongly supports the biological relevance of these metabolic processes, particularly the coordinated regulation of hypoxia adaptation through HIF‐1 signaling [25, 26]. These findings not only conform to the known biological characteristics of ccRCC but also expand our understanding of metabolic vulnerability. MR IVW analysis provides strong evidence for lipid metabolism disorders as causal risk factors for the development of ccRCC, and the consistency of symmetric funnel plots and MR Egger/IVW estimates confirms the reliability of the results. This causal relationship emphasizes the importance of metabolic intervention in the prevention and treatment of ccRCC. MR IVW analysis provides genetic evidence for lipid metabolism disorders as a risk factor for the development of ccRCC, and the consistency of symmetric funnel plots and MR Egger/IVW estimates validates the reliability of the results. However, MR analysis has its own limitations, including potential residual pleiotropy and inability to distinguish between direct and indirect effects. Therefore, further functional validation research is needed to verify this causal relationship.

Our prognostic model construction combines traditional statistical methods (univariate/multivariate Cox regression) with advanced machine learning techniques (SHAP analysis). The forest map of 86 prognostic genes and subsequent determination of SUCLG1, SUCLA2, ACAT1 and HMGCS2 as the main contributing factors for risk prediction based on SHAP values. Among them, the role of SUCLG1 in tumors is through metabolic reprogramming, which converts the metabolite signal of succinyl CoA into instructions for regulating mitochondrial gene expression in ccRCC, and low expression of SUCLG1 may promote malignant progression of cancer by disrupting mitochondrial function [23, 27]. Similarly, the core function of SUCLA2 is to serve as a key regulatory node for glutamine metabolism, precisely controlling the activity of GLS through protein interactions and posttranslational modifications in ccRCC with low expression of SUCLA2; tumor cells may be particularly sensitive to GLS inhibitors because they lack this intrinsic regulatory mechanism and rely more on the sustained activity of GLS [28]. ACAT1 is a key enzyme involved in ketone body metabolism and cholesterol metabolism in mitochondria, and is crucial for maintaining mitochondrial lipid homeostasis. Its low expression promotes malignant progression of tumors by disrupting mitochondrial lipid metabolism homeostasis [29, 30]. In addition, HMGCS2 is the rate limiting enzyme in the process of ketone body formation, and its low expression leads to the closure of this normal metabolic pathway. The inactivation of this critical metabolic node may force cancer cells to switch to other energy sources and alter their lipid utilization patterns, thereby supporting their rapid proliferation [21]. It is interesting that pyruvate carboxylase (PC) is considered an oncogene in most cancers, and in the specific cancer type ccRCC, we speculate that PC may play a role as a tumor suppressor gene or metabolic adaptive limiting factor. Its low expression promotes malignant progression of tumors by altering the distribution of metabolic flux [24, 31]. The directional consistency between multiple gene expression changes and SHAP values enhances the biological interpretability of the model. It is worth noting that the predictive accuracy of this model is superior to conventional clinical parameters, and its clinical applicability has been demonstrated through validation in multiple analysis methods (PCA/UMAP/t‐SNE) and independent cohorts (TCGA/GEO). In parallel with our findings, a recent multi‐omics and machine learning study by Chen et al. identified ABCG2 as a therapeutic target of Eleven Flavored Shenqi Tablets (EFST) in ccRCC, demonstrating that EFST‐associated genes exhibit high predictive capacity and that ABCG2 promotes ccRCC proliferation and migration. This independent study further supports the utility of multi‐omics and machine learning approaches for discovering novel therapeutic targets and prognostic signatures in ccRCC [32].

A comprehensive analysis of immune characteristics revealed different microenvironmental features among risk groups. Low‐risk tumors exhibit preserved immune surveillance function (resting CD4+ memory T cells, monocytes, and resting dendritic cells) coexisting with elevated M0/M2 macrophages, suggesting dynamic myeloid plasticity. In contrast, high‐risk tumors exhibit significant immune suppression (Tfh and Tregs aggregation) accompanied by impaired cell lysis activity—these findings are associated with the metabolic drivers identified by our SHAP through lactate‐mediated immune suppression mechanisms. Immunotyping further confirmed these differences. These results provide a biological basis for the differential immune therapy response observed in clinical practice.

Genomic analysis revealed significant mutation differences, particularly in high‐risk tumors where TMB elevation coexists with its immunosuppressive phenotype. This seemingly contradictory phenomenon may reflect the immune escape mechanism driven by genomic instability, and the elevated TIDE score supports this viewpoint. Drug sensitivity analysis identified treatment options for risk stratification, with LY2109761 (TGF‐β inhibitor) and carmustine (alkylating agent) showing particularly promising outcomes for high‐risk patients (Cor > 0.17, *p* < 0.001) [33, 34]. Similarly, recent studies have identified additional therapeutic candidates for RCC, including oxymatrine, which suppresses RCC progression by increasing TOR1AIP1 expression and inactivating the JNK signaling pathway [35]. The cross‐risk efficacy of temozolomide, MIRA‐1, and EPZ004777 suggests their potential as broad‐spectrum therapeutic agents for ccRCC.

In summary, the five core genes exhibit a unified expression and prognostic pattern in ccRCC: they all serve as potential tumor suppressor genes, with significantly downregulated expression levels and negatively correlated with adverse prognostic indicators such as high tumor staging and low survival rate. We speculate that these five genes together form a metabolic reprogramming network that inhibits the development of ccRCC, and their functional inactivation may synergistically drive the malignant progression of tumors by disrupting key processes such as lipid, amino acid, or energy metabolism. These results are consistent at the transcriptional and protein validation levels, emphasizing the interaction between genetic markers and established clinical prognostic factors.

Finally, we investigated the potential physiological role of SUCLA2 in the initiation and progression of ccRCC. Our functional experiments demonstrated that knockdown of SUCLA2 significantly enhanced the proliferative and invasive capacities of ccRCC cells, while its overexpression suppressed these malignant phenotypes. These findings suggest that SUCLA2 may function as a tumor suppressor in ccRCC, and its downregulation could contribute to tumor aggressiveness. Given its role as a key metabolic enzyme involved in the TCA cycle and glutamine metabolism, SUCLA2 loss may disrupt mitochondrial energy homeostasis and promote metabolic reprogramming, thereby facilitating ccRCC progression. Although the precise molecular mechanisms remain to be elucidated, our results highlight the therapeutic potential of targeting SUCLA2 or its downstream pathways in ccRCC. Future studies are warranted to dissect the underlying signaling networks and validate SUCLA2 as a viable target for clinical intervention. Compared to the recent study by Liu et al. [36], which constructed a 17‐gene metabolic signature using single‐cell RNA‐seq and machine learning, our study distinguishes itself in three aspects. First, in bioinformatics analysis, we integrated multi‐omics data (transcriptomics, genomics, and pharmacogenomics) with MR to establish causality between lipid metabolism disorders and ccRCC risk, whereas their analysis was primarily correlational. Second, in experimental validation, while they focused solely on GGT6 knockdown, we systematically validated all five core genes at both mRNA and protein levels, and performed bidirectional functional assays (overexpression and knockdown) for SUCLA2, providing the first causal evidence of its tumor‐suppressive role in ccRCC. Third, in overall research strategy, our study integrates multi‐omics discovery, causal inference, immune microenvironment dissection, drug sensitivity profiling, and comprehensive experimental validation, establishing a more holistic and clinically translatable precision oncology framework. This study establishes metabolic reprogramming as a core regulatory mechanism in ccRCC progression. Through systematic bioinformatics analysis, we established an exploratory prognostic prediction model and revealed potential risk‐stratified therapeutic targets. However, these findings require validation in large prospective cohorts and clinical trials before being applied to clinical risk stratification or treatment selection. The proposed five‐gene signature should be considered a hypothesis‐generating tool rather than a clinically ready assay.

Several limitations need to be considered: Firstly, although our model demonstrates strong predictive performance, prospective clinical validation is still needed. Secondly, the exact mechanism of specific metabolic genes and immune regulation needs to be experimentally validated. Thirdly, CIBERSORT and ESTIMATE are both computational prediction algorithms based on transcriptome expression profiles, and their results reflect relative abundance or scores rather than absolute cell counts or direct histological measurements. The results of immune infiltration analysis should be regarded as a biological hypothesis of computer simulation, rather than a substitute for experimental verification. The drug sensitivity analysis in this study is based on the IC_50_ values predicted by a computational model trained on the GDSC2 cell line drug response database, reflecting the inherent sensitivity prediction of tumor cell lines to drugs, rather than actual drug response data from patient tumor tissues. The screened compounds (such as LY2109761 and carmustine) should be considered as priority candidate drugs for validation, and their clinical translational value needs to be evaluated in future prospective clinical trials and/or patient derived xenograft models.

## 5 Conclusion

This multi‐omics study established metabolic reprogramming as the core regulatory mechanism for the progression of ccRCC, integrating the intrinsic metabolic changes of tumors with the reshaping of the external immune microenvironment. A robust prognostic prediction model was established and validated through systematic bioinformatics analyses and machine learning methods, revealing risk‐stratification therapy targets with direct clinical value. The discovery of drug sensitivity patterns provides a potential clinical translational treatment pathway for clinical practice. Fundamentally advancing the understanding of ccRCC as a “metabolic immune disorder” disease, and providing a theoretical framework for developing precision medicine strategies that simultaneously target these two biological axes.

## Author Contributions

**Yi Peng:** writing – review and editing, writing – original draft, conceptualization, methodology, data curation, visualization. **Suobing Qin:** writing – original draft, validation, formal analysis. **Xiwen Li:** investigation, software. **Lijun Wan:** writing – original draft, validation, investigation. **Rijian Guan:** software, data curation, supervision.

## Funding

The authors have nothing to report.

## Ethics Statement

The authors have nothing to report.

## Consent

The authors have nothing to report.

## Conflicts of Interest

The authors declare no conflicts of interest.

## Supporting information


**Table S1:** The primer sequences for five core genes and internal references.
**Table S2:** The antibody information.
**Table S3:** The causal relationship between lipid metabolism disorders and ccRCC.

## Data Availability

The data that support the findings of this study are available from the corresponding author upon reasonable request.
