# Correction to “Multi‐Omics Integration Identifies a Five‐Gene Metabolic Signature With Experimental Validation in Clear Cell Renal Cell Carcinoma”

**DOI:** 10.1002/cnr2.70724

**Published:** 2026-10-18

Y. Peng , S. Qin , X. Li , L. Wan , and R. Guan , “Multi‐Omics Integration Identifies a Five‐Gene Metabolic Signature With Experimental Validation in Clear Cell Renal Cell Carcinoma,” Cancer Reports 9, no. 9 (2026): e70673, 10.1002/cnr2.70673.PMC1354774342702836

In the abovementioned article, there was an error in Figure 10D, where the ACAT1 and HMGCS2 bands were inadvertently duplicated. This error does not affect the results or conclusions of the study.

The corrected Figure 10D is provided below:

**FIGURE 10:**
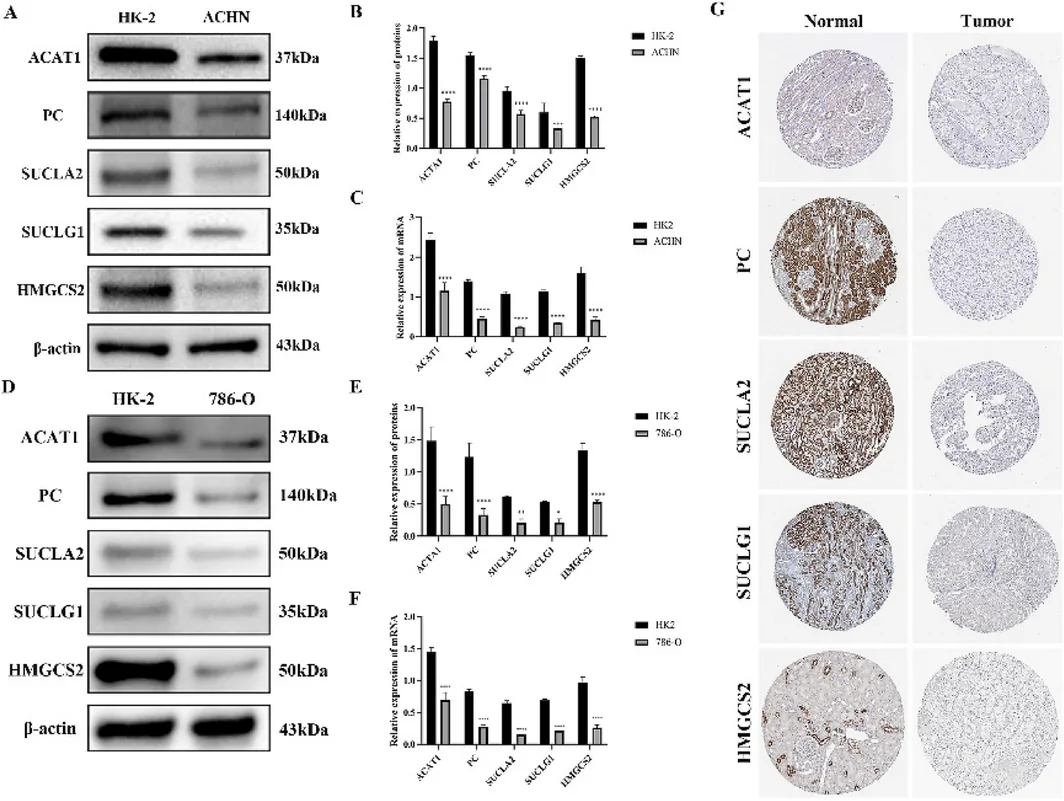
Validation of protein and mRNA expression levels of the five core genes. Immunohistochemical images from the Human Protein Atlas (HPA) database, quantitative PCR, and western blot results confirm the protein and transcriptional expression patterns of (A–C) protein expression levels of the five core genes in the ACHN cell line. (B–F) mRNA expression levels of the five core genes in the 786‐O cell line. (G) Immunohistochemical images of the five core genes. *p < 0.05; **p < 0.01; ***p < 0.001; ****p < 0.0001.

We apologize for this error.

